# *Pinus densiflora* Sieb. et Zucc. Alleviates Lipogenesis and Oxidative Stress during Oleic Acid-Induced Steatosis in HepG2 Cells

**DOI:** 10.3390/nu6072956

**Published:** 2014-07-23

**Authors:** Yu-Jin Hwang, Hae-Ri Wi, Haeng-Ran Kim, Kye Won Park, Kyung-A Hwang

**Affiliations:** 1 Department of Agrofood Resources, National Academy of Agricultural Science, RDA, Suwon 441-853, Korea; E-Mails: yujinh21@skku.edu (Y.-J.H.); hr226@naver.com (H.-R.W.); kimhrr@korea.kr (H.-R.K.); 2 Department of Food Science and Biotechnology, Sungkyunkwan University, Suwon 440-746, Korea; E-Mail: kwpark@skku.edu

**Keywords:** *Pinus densiflora* Sieb. et Zucc., nonalcoholic fatty liver disease, hepatic lipid accumulation, oxidative stress

## Abstract

Excess accumulation of lipids and oxidative stress in the liver contribute to nonalcoholic fatty liver disease (NAFLD). We hypothesized that *Pinus densiflora* Sieb. et Zucc. (PSZ) can protect against NAFLD by regulating lipid accumulation and oxidative stress in the liver. To investigate the effect of PSZ upon NAFLD, we used an established cellular model: HepG2 cells treated with oleic acid. Then, the extent of hepatic steatosis and oxidative stress was assessed and levels of inflammatory markers measured. Oleic acid-treated HepG2 cells, compared with controls, had greater lipid accumulation. PSZ decreased lipid accumulation by 63% in oleic acid-treated HepG2 cells. Additionally, PSZ decreased the target gene expression of lipogenesis such as sterol regulatory element binding protein-1c, fatty acid synthase, stearoyl-CoA desaturase-1, diacylglycerol *O*-acyltransferase-1, and acetyl-CoA carboxylase-1 by 1.75, 6.0, 2.32, 1.93 and 1.81 fold, respectively. In addition, Oleic acid-treated HepG2 cells elicited extensive accumulation of tumor necrosis factor-α (TNFα) by 4.53 fold, whereas PSZ-treated cells decreased the expression of TNFα mRNA by 1.76 fold. PSZ significantly inhibited oxidative stress induced by reactive oxygen species. These results suggest that PSZ has effects on steatosis *in vitro* and further studies are needed *in vivo* to verify the current observations.

## 1. Introduction

The liver is the main organ for coordinating regulation of energy metabolism and lipid trafficking. Excess intake of lipids in the diet can lead to fatty liver, which can progress to nonalcoholic fatty liver disease (NAFLD) and non-alcoholic steatohepatitis (NASH). NAFLD is defined as hepatic macrovesicular steatosis upon consumption of alcohol of <20 g/day. It is the most common liver disease in the USA [1,2,3,4]. NAFLD encompasses a wide spectrum of disorder and damage to the liver characterized by hepatic steatosis without significant consumption of alcohol and includes simple steatosis, inflammatory NASH, liver cirrhosis, and hepatocellular carcinoma [5].

Disorders of lipid metabolism in the liver are likely to play important parts in the initiation and development of NAFLD [6]. According to the “two hit’’ pathophysiological theory, the “first hit” is deposition of free fatty acids and triglyceride in hepatocytes (*i.e.*, steatosis). The “second hit” is the progression of steatosis to NASH. This progression is associated with oxidative stress and the release of cytokines that can induce inflammation, fibrosis, or necrosis [7]. Further, the pro-inflammatory cytokine tumor necrosis factor (TNF)-α has a central role in the etiology of NASH due to its stimulation of hepatic lipogenesis and adipose lipolysis [8].

Oxidative stress is thought to be the main factor involved in the etiology of NASH. Oxidative stress results from an imbalance between pro-oxidant and antioxidant chemical species that leads to oxidative damage to cellular macromolecules [9]. The predominant pro-oxidant chemicals in fatty livers are singlet oxygen molecules, superoxide anions, hydrogen peroxide, and hydroxyl radicals: molecules referred to collectively as “reactive oxygen species” (ROS). Oxidation of fatty acids is an important source of ROS in fatty livers [10,11,12,13]. Thus, agents that can prevent or attenuate free fatty acid-induced lipogenesis and oxidative stress-induced damage represent promising therapeutic choices for NAFLD. Several agents are being tested for the treatment of NAFLD and, recently, the use of plants has emerged as a possible means to alleviate NAFLD. However, no medication has been approved for treating NAFLD [14,15,16].

*Pinus densiflora* Sieb. et Zucc. (PSZ) has been used as a folk remedy for rheumatism, hemorrhage, gastroenteritis, hypertension and asthma [17]. Recently, a broad spectrum of beneficial effects (antioxidative [18], anti-inflammatory [19,20], antitumor [21]) of PSZ has been reported. However, the anti-NAFLD effects of PSZ have not been identified. According to Kim [22], PSZ has several phytochemicals such as α-pinene, β-pinene, camphene, quercetin, kaempferol, ρ-coumaric acid and choline. The biological or therapeutic activities of medicinal plants are closely related to their chemical compounds. For example, ρ-coumaric acid was found to modulate lipid metabolism in HepG2 cells via the AMPK activation pathway [23]. Quercetin effectively reversed NAFLD symptoms by decreased triacylglycerol accumulation, insulin resistance, inflammatory cytokine secretion and increased cellular antioxidants in OA induced hepatic steatosis in HepG2 cells [24]. In addition, due to NAFLD induced by choline-deficient, choline-rich PSZ would be help to improve NAFLD [25]. Often, the combination of several components is more effective than using any single constituent alone. Therefore, due to the synergistic effect of known components such as ρ-coumaric acid, choline and quercetin, PSZ would be useful material for the anti-NAFLD.

In the present study, we investigated the beneficial effects of PSZ (a candidate for the treatment of NAFLD) on the inhibition of fat accumulation and oxidative stress in the liver.

## 2. Experimental Section

### 2.1. Samples, Antibodies, and Reagents

Ethanol (70%) extracts of PSZ was purchased from the Plant Extract Bank (Je-ju, Korea). Dulbecco’s modified Eagle’s medium (DMEM), fetal bovine serum (FBS), and penicillin-streptomycin were obtained from Gibco (Carlsbad, CA, USA). Antibodies against sterol regulatory element binding protein-1c (SREBP-1c), liver X receptor-α (LXRα), 5′ AMP-activated protein kinase (AMPK) and β-actin were purchased from Abcam (Cambridge, MA, USA). Oil-red-O, oleic acid (OA) was obtained from Sigma-Aldrich (Saint Louis, MO, USA). Nile Red, boron-dipyrromethene (BODIPY 493/503) and dichlorofluorescein diacetate (DCF-DA) were purchased from Invitrogen (Carlsbad, CA, USA). CellTiter Glo was obtained from Promega (Madison WI, USA). All other chemicals were purchased from Sigma-Aldrich unless specified otherwise.

### 2.2. OA/BSA Complex Solution Preparation

OA/BSA complex solution was prepared by a slight modification of previously described methods [26]. One hundred mM OA stock solution was prepared in 0.1 N NaOH by heating at 70 °C in a shaking water bath. In an adjacent water bath at 55 °C, a 10% (w/v) FFA-free BSA solution was prepared in H_2_O. Twenty mM OA containing 10% BSA was diluted in the culture medium to obtain the desired final concentrations. The OA/BSA complex solution was sterile-filtered through a 0.45 μm pore membrane filter and stored at −20 °C.

### 2.3. Cell Culture

The human hepatocellular carcinoma cell line HepG2 was obtained from Korean Cell Line Bank (Seoul, Korea). HepG2 cells were routinely cultured in DMEM (Gibco) supplemented with 10% FBS and 1% penicillin-streptomycin in an incubator under an atmosphere of 5% CO2 at 37 °C. To accumulate fatty acids, HepG2 cells were exposed to OA at a final concentration of 2 mM for 24 h.

### 2.4. Cytotoxicity

HepG2 cells (1 × 10^5^ cells/well) in 24-well plates were treated with PSZ. PSZ ethanol extract in dimethyl sulfoxide (DMSO) was diluted in phosphate-buffered saline (PBS) to obtain final concentrations of 100, 200, and 500 μg/mL. Cells were treated with extract samples for 24 h, and cell viability measured with CellTiter Glo^®^ (Promega). Viability is presented as the percentage of live cells in each well.

### 2.5. Staining Using Oil-red-O, BODIPY, and Nile Red

HepG2 cells (2 × 10^5^ cells/mL) were treated with PSZ (100 μg/mL) and OA (2 mM) for 24 h. Resveratrol (50 μM) as the one of effective natural compounds is used as the positive control. After incubation, cells were fixed with 4% paraformaldehyde and stained with a freshly prepared working solution of Oil-red-O for 20 min at room temperature. After several washings, cells were observed under a microscope (Nikon, Tokyo, Japan).

To quantify Oil-red-O content, isopropanol was added to each sample, which was shaken at room temperature for 5 min, and the optical density of the isopropanol-extracted sample read using a spectrophotometer at 510 nm.

### 2.6. Real-Time Reverse Transcription-Polymerase Chain Reaction (RT-PCR) Analyses

RT-PCR was undertaken to determine the expression of lipids using a Rotor-Gene Q Real-time Thermal Cycler (Qiagen, Stanford, VA, USA) according to manufacturer instructions. HepG2 cells were treated with PSZ (100 μg/mL) and OA (2 mM) for 24 h. After incubation, cDNA was synthesized from the total RNA isolated from cells. The PCR was carried out using 2X SYBR Green mix (Qiagen). All results were normalized to expression of glyceraldehydes-3-phosphate dehydrogenase. Primer sequences are shown in Table 1.

**Table 1:** Gene-specific primers used for real-time RT-PCR.

Gene	Forward	Reverse
SREBP-1	5′-GCGGAGCCATGGATTGCAC-3′	5′-TCTTCCTTGATACCAGGCCC-3′
FAS	5′-AGCTGCCAGAGTCGGAGAAC-3′	5′-TGTAGCCCACGAGTGTCTCG-3′
SCD1	5′-CCAACACAATGGCATTCCAG-3′	5′-GGTGGTCACGAGCCCATTC-3′
PPARγ	5′-GAACAGATCCAGTGGTTGCAG-3′	5′-GGCATTATGAGACATCCCCAC-3′
DGAT1	5′-GGCATCCTGAACTGGTGTGTG-3′	5′-GAGCTTGAGGAAGAGGATGGTG-3′
ACC1	5′-GAGGGCTAGGTCTTTCTGGAAG-3′	5′-CCACAGTGAAATCTCGTTGAGA-3′
PPARα	5′-TCCGACTCCGTCTTCTTGAT-3′	5′-GCCTAAGGAAACCGTTCTGTG-3′
CPT1	5′-TGAGCGACTGGTGGGAGGAG-3′	5′-GAGCCAGACCTTGAAGTAGCG-3′
ACOX	5′-TCCTGCCCACCTTGCTTCAC-3′	5′-TTGGGGCCGATGTCACCAAC-3′
ACC2	5′-GCCAGAAGCCCCCAAGAAAC-3′	5′-CGACATGCTCGGCCTCATAG-3′
TNFα	5′-CAGCCTCTTCTCCTTCCTGAT-3′	5′-GCCAGAGGGCTGATTAGAGA-3′

### 2.7. Western Blot Analyses

HepG2 cells were treated with PSZ (100 μg/mL) and OA (2 mM) for 24 h. After incubation, cells were lysed with lysis buffer (150 mM sodium chloride, 1% Triton X-100, 1% sodium deoxycholate, 0.1% sodium dodecyl sulfate, 50 mM Tris-HCl, pH 7.5, and 2 mM ethylenediamine tetra-acetic acid) on ice for 30 min. After centrifugation (12,000× *g*, for 15 min at 4 °C), the supernatant was collected, and protein concentrations determined using a bicinchoninic acid assay (GenDEPOT; Barker, TX, USA). Equal amounts (10 μg) of cell extracts were separated by sodium dodecyl sulfate-polyacrylamide gel electrophoresis and transferred to polyvinylidene difluoride membranes (Bio-Rad, Hercules, CA, USA). Membranes were blotted with antibody, and detection undertaken with an enhanced chemiluminescence system (Pierce, Rockford, IL, USA) according to manufacturer instructions.

### 2.8. Cytokine Determinations

HepG2 cells were treated with PSZ (100 μg/mL) and OA (2 mM) for 24 h. After incubation, the supernatants were collected. The TNFα levels in the culture medium were determined by a Duo Set mouse TNFα ELISA kit according to the manufacturer's protocols (R&D Systems, Minneapolis, MN, USA).

### 2.9. Flow Cytometric Analyses of the Scavenging Activity of Intracellular ROS

HepG2 cells were treated with PSZ (100 μg/mL) and OA (2 mM) for 24 h. After incubation, cells were stained with dichlorofluorescein diacetate (DCF-DA) according to manufacturer instructions. Stained cells were subjected to flow cytometric analyses using FACSCalibur (Becton Dickinson Biosciences, San Jose, CA, USA).

### 2.10. Statistical Analyses

Statistical analyses were undertaken with SPSS v12.0 (SPSS, Chicago, IL, USA). Data are the mean ± SEM from three independent experiments unless stated otherwise. Statistical analyses were done using the Student’s *t*-test and *p* < 0.05 was considered significant.

## 3. Results

### 3.1. Cytotoxic Effects of PSZ on HepG2 Cells

**Figure 1:**
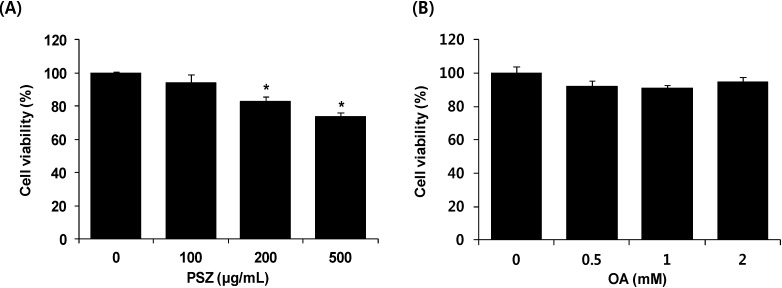
Cell viability effect of *Pinus densiflora* Sieb. et Zucc. (PSZ) in HepG2 cells. (**A**) HepG2 cells were treated with PSZ (100, 200 and 500 μg/mL) and (**B**) HepG2 cells were treated with oleic acid (OA; 0.5, 1 and 2 mM). After treatment for 24 h, cell viability was quantified by measuring intracellular levels of ATP. Bars represent the mean ± SEM of three experiments done in triplicate.

Cytotoxic effects of PSZ on HepG2 cells were determined by exposing them to PSZ (100, 200, and 500 μg/mL) for 24 h. We estimated the influence on cell survival according to the following criteria [27,28]: Cell viability values greater than 90% were considered unaffected by tested compounds, 80%–90% was modestly affected, and values less than 80% were considered affected by the cytotoxic effects of the compounds. PSZ suppressed the proliferation of HepG2 cells in a dose-dependent manner (Figure 1A). In groups treated with 200 and 500 μg/mL PSZ, the viability of HepG2 cells after exposure was 82% and 73%, respectively. HepG2 cells were treated with 0–2 mM OA for 24 h to induce hepatic steatosis, yet this action did not result in cytotoxicity (Figure 1B). Therefore, according to criteria, 100 μg/mL PSZ and 2 mM OA were used to induce fatty liver.

### 3.2. PSZ Reduces OA-Induced Steatosis in HepG2 Cells

**Figure 2:**
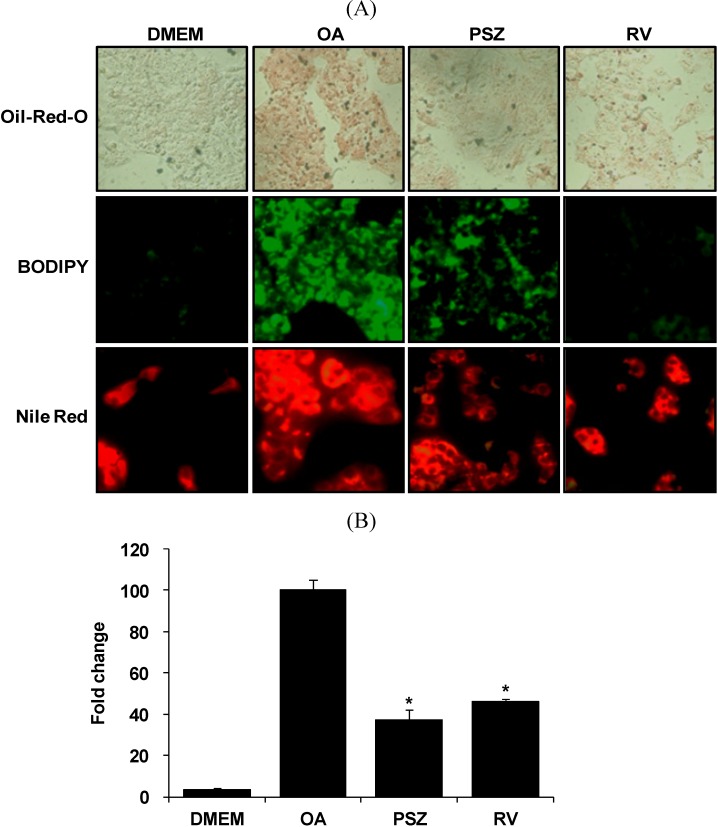
Effects of *Pinus densiflora* Sieb. et Zucc. (PSZ) on steatosis in HepG2 cells stimulated with oleic acid (OA). (**A**) HepG2 cells were treated with 100 μg/mL PSZ. After treatment for 24 h, lipid accumulation was measured by staining with Oil-red-O, BODIPY, and Nile Red. (**B**) Lipid accumulation was quantified by measuring the extracted dye at 510 nm. Results are the mean ± SEM from three independent experiments. * *p* < 0.05 compared with the OA group. DMEM, control group; OA, oleic acid-treated group; PSZ, OA + *Pinus densiflora* Sieb. et Zucc.*-*treated group; RV, OA + resveratrol-treated group.

Cultured HepG2 cells were exposed to OA and lipid accumulation detected by staining using Oil-red-O, BODIPY and Nile Red after 24 h. Significant differences between control groups and OA groups were noted (Figure 2A). HepG2 cells in the control group did not suffer steatosis, whereas cells in the OA group had severe steatosis. A greatly reduced number of lipid droplets was noted in the PSZ group compared with the OA group. Lipid accumulated in OA-treated cells and PSZ significantly decreased lipid accumulation by 63% compared with the OA group. Resveratrol reduced TG content by 54% compared with the OA group (Figure 2B).

We measured the lipid accumulation by various methods (Oil-red-O, BODIPY and Nile Red). The reason is to confirm that it is not just an Oil-red-O specific effect by using at least one other staining reagent. Whereas Oil-red-O was used a total lipid staining, the BODIPY and Nile Red fluorescence can be used to distinguish neutral lipids from phospholipids or other amphipathic lipids [29,30]. That is why the staining patterns of our result are different. The BODIPY and Nile Red dye reveals intensely staining foam cells and lipid droplet clusters, as well as a less intense widespread staining of almost the entire cell.

### 3.3. PSZ Attenuates Hepatic Steatosis via Inhibition of Expression of Lipogenic Genes

**Figure 3:**
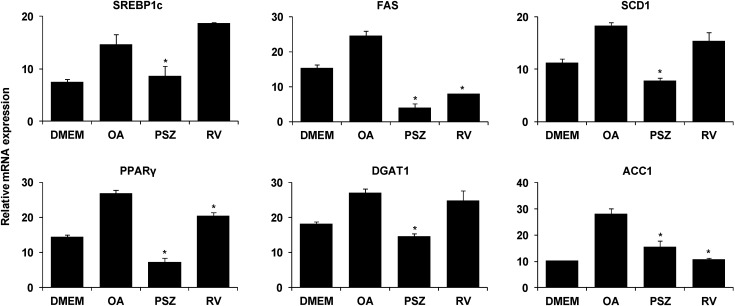
Effects of *Pinus densiflora* Sieb. et Zucc. (PSZ) on expression of lipogenesis genes in HepG2 cells stimulated with oleic acid (OA). HepG2 cells were treated with 100 μg/mL PSZ and OA. After treatment for 24 h, RNA was isolated and reverse-transcribed for RT-PCR analyses using the primers described in Table 1. Results are the mean ± SEM from three independent experiments. * *p* < 0.05 compared with the OA group. DMEM, control group; OA, oleic acid-treated group; PSZ, OA + *Pinus densiflora* Sieb. et Zucc.-treated group; RV, OA + resveratrol-treated group.

**Figure 4:**
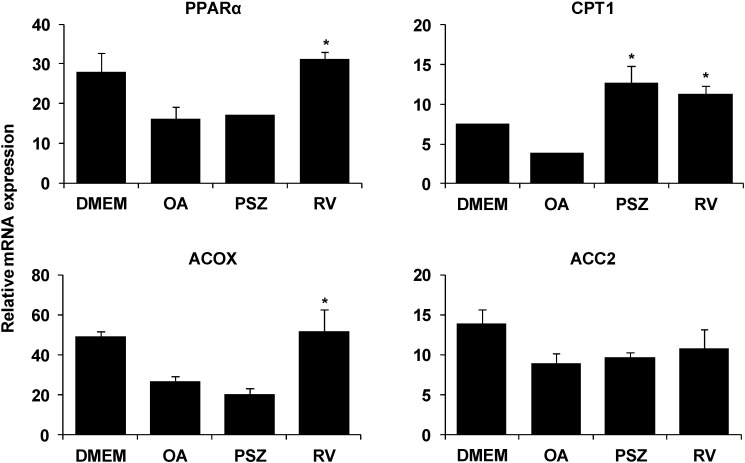
Effects of *Pinus densiflora* Sieb. et Zucc. (PSZ) on expression of hepatic lipolysis genesin HepG2 cells stimulated with oleic acid (OA). HepG2 cells were treated with 100 μg/mL PSZand OA. After treatment for 24 h, RNA was isolated and reverse-transcribed for RT-PCR analyses using the primers described in Table 1. Results are the mean ± SEM from three independent experiments. * *p* < 0.05 compared with the OA group. DMEM, control group; OA, oleic acid-treated group; PSZ, OA + *Pinus densiflora* Sieb. et Zucc.-treated group treated group; RV, OA + resveratrol-treated group.

We examined the expression of genes that regulate lipid metabolism to better define how PSZ inhibits hepatic steatosis. Expression of the lipogenic genes SREBP-1c, diacylglycerol-*O*-acyltransferase 1 (DGAT1), peroxisome proliferator-activated receptor gamma (PPARγ), stearoyl-CoA desaturase 1 (SCD1), fatty acid synthase (FAS), and acetyl-CoA carboxylase (ACC1) was upregulated significantly in OA-treated HepG2 cells. PSZ decreased the mRNA expression of lipogenic genes (Figure 3). In contrast, PSZ did not increase the expression of lipolytic genes such as PPARα, acyl-CoA oxidase (ACOX) and ACC2 except carnitine palmitoyltransferase I (CPT1) (Figure 4). We confirmed that resveratrol affected the lipolytic pathway rather than inhibiting the lipogenic pathway. Collectively, these data suggested that PSZ suppressed hepatic lipogenesis and CPT1-mediated lipolysis.

### 3.4. PSZ Attenuates the Transcription Factors for Lipogenic Genes

SREBP-1c is a transcription factor that regulates the expression of lipogenic genes, including ACCs and FAS. SREBP-1c is activated by LXR (a nuclear receptor that regulates the metabolism of cholesterol and fatty acids) [31]. AMPK is a sensor of energy homeostasis in cells. If AMPK is activated, it “switches on” catabolic pathways (e.g., fatty-acid oxidation and glycolysis) and “switches off” adenosine triphosphate-consuming pathways (e.g., lipogenesis) [9].

**Figure 5:**
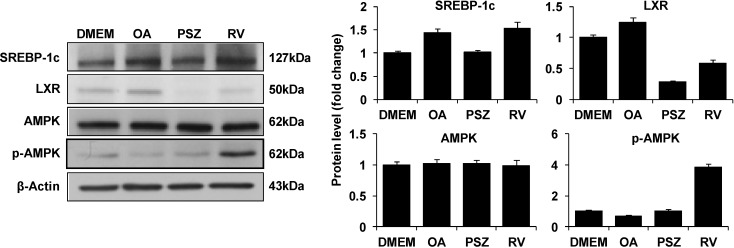
Effects of *Pinus densiflora* Sieb. et Zucc. (PSZ) on expression of the transcription factor for lipogenic genes in HepG2 cells stimulated with oleic acid (OA). HepG2 cells were treated with 100 μg/mL PSZ and OA. After treatment for 24 h, whole-cell lysates were subjected to western blot analyses for anti-SREBP-1c, LXR, and AMPK antibodies. β-Actin was used as the internal control. Results are the mean ± SEM from three independent experiments. DMEM, control group; OA, oleic acid-treated group; PSZ, OA + *Pinus densiflora* Sieb. et Zucc.-treated group; RV, OA + resveratrol-treated group.

To understand the mechanisms involved in the pathogenesis of NAFLD, we investigated expression of transcription factors by western blotting. The level of immature SREBP-1c increased in the OA-treated group of HepG2 cells (Figure 5). However, immature SREBP-1c expression decreased in cells treated with PSZ compared with that seen in the OA group. Similar results were obtained for LXR expression. Expression of AMPK protein was not significantly different in the PSZ group compared with that in the OA group. In addition, we found that the expression pattern of p-AMPK did not change in the PSZ-treated group but p-AMPK was increased in the RV group. Taken together, these data suggested that PSZ suppressed hepatic lipogenesis by regulating expression of LXR and SREBP-1c.

### 3.5. PSZ Decreases Hepatic Inflammation by Attenuating TNFα Expression

**Figure 6:**
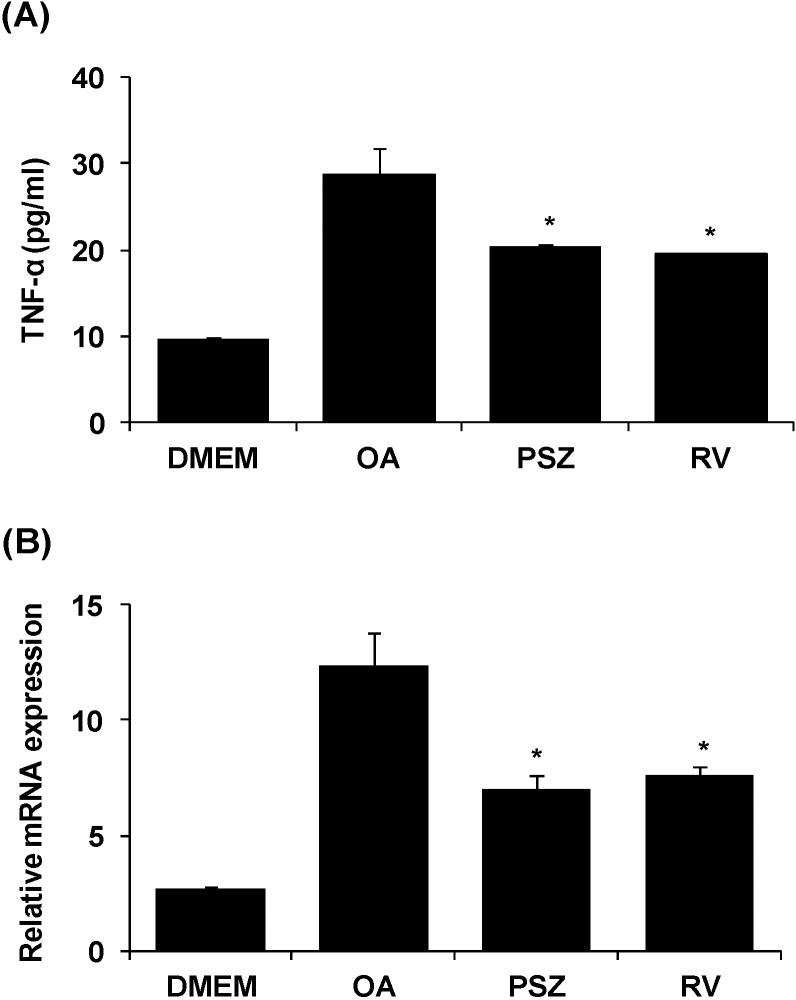
Effects of *Pinus densiflora* Sieb. et Zucc. (PSZ) on TNFα production (**A**) and mRNA expression (**B**) in oleic acid (OA)-induced HepG2 cells. (A) HepG2 cells were treated with 100 μg/mL PSZ. After treatment for 24 h, the supernatant was collected. TNFα production was quantified by ELISA. (B) HepG2 cells were treated with 100 μg/mL PSZ. After treatment for 24 h, RNA was isolated and reverse-transcribed for RT-PCR analyses using the primers described in Table 1. Results are the mean ± SEM from three independent experiments. * *p* < 0.05 compared with the OA group. DMEM, control group; OA, oleic acid-treated group; PSZ, OA + *Pinus densiflora* Sieb. et Zucc.*-*treated group; RV, OA + resveratrol treated group.

Accumulation of FFA results in lysosomal permeabilization and then release of a lysosomal protease, namely ctsb, into the cytosol activates a feature of TNFα signaling cascades [32]. Hepatic steatosis is associated with increased expression of TNFα, which stimulates lipogenesis and lipolysis as well as inducing hepatic dysfunction [33]. We confirmed TNFα production by the enzyme-linked immunosorbent assay (ELISA). TNFα content was greater in the OA group and lower in the OA + PSZ group (Figure 6A). Expression of TNF-α mRNA was six-fold greater in the OA group and was decreased by 43% in the OA + PSZ (Figure 6B).

### 3.6. PSZ Inhibits ROS Production

**Figure 7:**
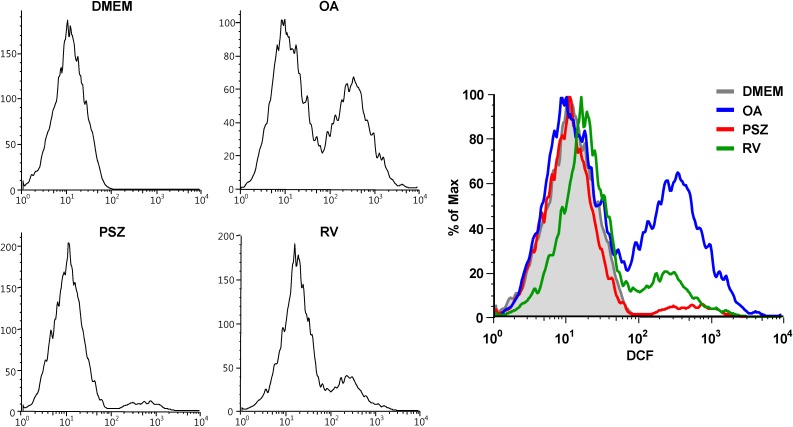
Effects of *Pinus densiflora* Sieb. et Zucc. (PSZ) on ROS-scavenging activity in oleic acid (OA)-induced HepG2 cells. HepG2 cells were treated with 100 μg/mL PSZ. After treatment for 24 h, cells were harvested and stained with DCF-DA. Results are the mean ± SEM from three independent experiments. * *p* < 0.05 compared with the OA group. DMEM, control group; OA, oleic acid-treated group; PSZ, OA + *Pinus densiflora* Sieb. et Zucc.-treated group; RV, OA + resveratrol-treated group.

NAFLD and oxidative stress are positively correlated, and the production of ROS is enhanced by excess fat accumulation [34]. Intake of large amounts of glucose and free fatty acids induces acetyl-CoA production and excess NADH. Accumulation of excess NADH causes a generation of ROS [35]. In addition, the increased TNFα by OA induce the expression of NOX3 protein and subsequently the induced NOX3 increase the ROS production [36]. The accumulated ROS generates a lipid peroxidation result in subsequent damage to hepatic membranes, proteins and DNA [37]. Therefore, we evaluated the inhibition of ROS production by PSZ on OA-induced HepG2 cell. OA treatment significantly increased ROS formation in HepG2 cells as determined by DCF-DA fluorescence (Figure 7). However, treatment with PSZ and resveratrol blocked OA-induced ROS generation.

## 4. Discussion

NAFLD is a clinicopathological change characterized by accumulation of triglycerides in hepatocytes. NAFLD is one of the most frequent causes of abnormal liver function tests [38]. Recently, the incidence of NAFLD has increased markedly, accompanying the increased prevalence of obesity and type-2 diabetes mellitus. More than 10% of patients with NAFLD progress to NASH, which is characterized by infiltration of inflammatory cells in the liver and the ballooning of hepatocytes. Liver cirrhosis and hepatocellular carcinoma eventually occur in some patients with NASH [39].

Several agents have been developed to improve NAFLD. In addition, due to problems related to conventional treatment methods and the side effects of these agents, research has concentrated on new treatments, such as using natural products, to develop treatments for NAFLD and NASH [40,41]. Many plants with different pharmacological features are known to contain chemical compounds that may be suitable for the treatment or prevention of NAFLD [42]. Several plants have been examined to identify natural products that could prevent hepatic lipogenesis and be new effective compounds in the treatment of NAFLD [43,44].

**Figure 8:**
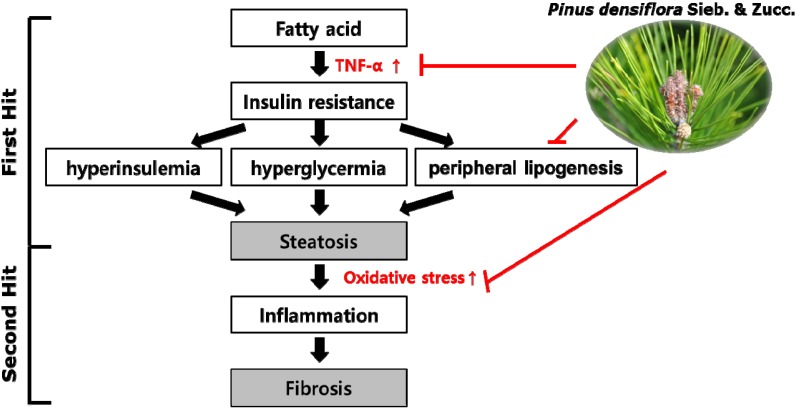
Proposed model for the inhibition of steatosis by *Pinus densiflora* Sieb. et Zucc. (PSZ) extracts in HepG2 cells. PSZ decreases TNF production, hepatic lipogenesis and oxidative stress, resulting in the improvement of hepatic steatosis.

We investigated if PSZ protects against NAFLD by decreasing lipogenesis. Our results also demonstrated that PSZ decreases the oxidative stress and inflammatory responses known to contribute to the development of NAFLD. In addition, PSZ inhibited the expression of TNFα protein and mRNA that are otherwise increased by NAFLD. Taken together, these results suggest that PSZ alleviates NAFLD by inhibiting hepatic lipogenesis by regulating expression of SREBP-1c and LXR as well as decreasing ROS production. Based on the results of the present study, the mechanism by which PSZ extract inhibits NAFLD in HepG2 cells is summarized in Figure 8.

This is the first study to demonstrate PSZ-mediated reductions in hepatic lipid accumulation. In OA-treated HepG2 cells, expression of lipogenic genes such as SREBP-1c, FAS and DGAT1 was upregulated. PSZ suppressed expression of these lipogenic genes, whereas it did not affect expression of lipolytic genes apart from CPT1. Lipid accumulation in liver may be caused by enhanced *de novo* lipogenesis, activation of lipid uptake, and lowering of lipid catabolism [45]. FAS, SCD-1 and DGAT1 are key enzymes in *de novo* fatty acid and TG synthesis in mammals. SREBP-1 is well known as the transcription factor regulating the gene expression of these lipogenic enzymes in the liver. FAS, SCD-1 and DGAT1 are known to be changed by SREBP-1 [46]. Our results indicate that the effect of PSZ on OA-induced hepatic lipogenesis in HepG2 cells is associated with decreased expression of SREBP-1 and its downstream lipogenic genes such as FAS, SCD-1 and DGAT1. Taken together, the decrease of FAS, SCD-1 and DGAT1 by regulation of SREBP-1 would be helpful to the inhibition of lipid accumulation by inhibiting the TG synthesis.

AMPK is involved in regulating hepatic lipogenesis and may be a therapeutic target for treating fatty liver disease [47]. It is known that SREBP-1c is negatively regulated by AMPK [48]. In the present study, it was found that OA significantly increased SREBP-1c and FAS expression in parallel with AMPK phosphorylation was decreased, indicating that the negative feedback regulation of SREBP-1c via AMPK in OA-induced conditions. PSZ also did not rescue the AMPK expression and the expression of AMPK target genes such as PPARα, ACOX, ACC2 except CPT1. In case of an increase in the CPT1 expression, PSZ causes inhibition of ACC1, which reduce the production of malonyl-CoA, an inhibitor of CPT1 [49,50]. Thus, ACC1 inhibition by PSZ appears to be important in regulating CPT1 activity. The results suggest that PSZ may also reduce lipid levels via promotion of hepatic fatty acid oxidation.

Various studies have shown that elevated TNFα plays a key role in the pathogenesis and disease progression of NAFLD [51,52]. According to Cui *et al.* [53], they reported that treatment of HepG2 cells with FFA resulted in increased the expression of TNF-α mRNA. In present study, we also confirm that OA treatment significantly increased TNF-α production from HepG2 cell. A key question is whether increased TNF-α in NAFLD is from hepatocytes or other inflammatory cells [54]. However, there should be further study to clarify whether the FFA-induced steatosis promotes expression of TNFα mRNA.

The present study showed that PSZ decreased TNFα production that was associated with increases in hepatic lipid content. In addition, NAFLD is also associated with oxidative stress and inflammatory responses that exacerbate liver injury [55]. Extracts from pine trees have been suggested to protect against oxidative stress [18]. Kwak *et al.* [21] reported that the free radical scavenging activity of pine needles ethanol extract appeared to be similar to that of α-tocopherol; the EC_50_ assessed for both pine needles ethanol extract and α-tocopherol was 95.12 μg/mL and 95.54 μg/mL, respectively. This result indicates that PSZ is very powerful antioxidant material. Also, according to Jung *et al.* [56], PSZ methanol extract and its various fractions have significant effects on scavenging free radicals. Our results showed that OA-treated HepG2 cells had elevated levels of ROS, whereas PSZ restored ROS to normal levels. This result is in agreement with their studies. However, additional *in vivo* work is needed to identify the exact antioxidant defense mechanism of PSZ extract.

## 5. Conclusions

PSZ effectively reversed NAFLD symptoms by decreasing lipid accumulation, secretion of inflammatory cytokines and oxidative stress in HepG2 cells. Hence, more experimental and clinical studies are needed to understand how PSZ prevents NAFLD.
